# Magnesium Hydroxide Nanoparticles Improve the Ocular Hypotensive Effect of Twice Daily Topical Timolol Maleate in Healthy Dogs

**DOI:** 10.3390/vetsci8080168

**Published:** 2021-08-23

**Authors:** Mizuki Kita, Kazutaka Kanai, Hiroki Mitsuhashi, Tomoki Noguchi, Noriaki Nagai, Mizuki Yamaguchi, Yuya Otaka, Rina Kudo, Yohei Yamashita, Kazuki Tajima

**Affiliations:** 1 Department of Small Animal Internal Medicine II, School of Veterinary Medicine, Kitasato University, 35-1 Higashi 23 ban-cho, Towada 034-8628, Aomori, Japan; dv18003@st.kitasato-u.ac.jp (M.K.); vm15126g@st.kitasato-u.ac.jp (H.M.); vm14100k@st.kitasato-u.ac.jp (T.N.); dv20001@st.kitasato-u.ac.jp (Y.O.); vm16502@st.kitasato-u.ac.jp (R.K.); ebisu2101@yahoo.co.jp (Y.Y.); tajima@vmas.kitasato-u.ac.jp (K.T.); 2 Faculty of Pharmacy, Kindai University, 3-4-1 Kowakae, Higashiosaka 577-8502, Osaka, Japan; nagai_n@phar.kindai.ac.jp (N.N.); 2033420005s@kindai.ac.jp (M.Y.)

**Keywords:** dogs, drug delivery system, glaucoma, magnesium hydroxide nanoparticles, timolol maleate

## Abstract

Timolol maleate (TM), a beta-adrenergic receptor antagonist, is widely used for canine antiglaucoma eye drops; however, its bioavailability is <5%. Our previous study revealed that magnesium hydroxide nanoparticles (nMH) have potency in improving the bioavailability of fixed-combined TM in rodent models. This study aimed to investigate whether the fixed combination with nMH improves the ocular hypotensive effect of TM and affects pupil size (PS), heart rate (HR), and mean arterial pressure (MAP) in clinically healthy dogs. Five clinically healthy dogs were administered topical saline, commercial 0.5% TM, and a 0.01% or 0.1% nMH–0.5% TM fixed combination (0.01% or 0.1% nMH–TM) twice daily in one eye for 7 days with at least a 28-day interval. The changes from baseline were calculated and were statistically analyzed for each drug. IOP was significantly reduced in both 0.01% and 0.1% nMH–TM-treated-dogs compared with saline- and TM-treated dogs. Meanwhile, 0.01% and 0.1% nMH did not exacerbate the side effects of TM. From these results, nMH improved the ocular hypotensive effect of TM without enhancing side effects. Topical nMH–TM is potentially more effective for canine ocular hypotensive eye drops than TM.

## 1. Introduction

Glaucoma is an ocular disease characterized by the progressive death of retinal ganglion cells and their axons [1,2,3,4]. There are a variety of risk factors for glaucoma, but an important modifiable risk factor is intraocular pressure (IOP) [1,2,4,5], especially in dogs [1,4]. Therefore, the mainstay of medical glaucoma therapy is aimed at reducing IOP, and it is the only evidence-based treatment approved for use in both dogs and humans [1,2,3,4,5,6].

Currently, there are a numerous commercially available antiglaucoma eye drops, including beta-adrenergic receptor antagonists, miotics, carbonic anhydrase inhibitors, and prostaglandin analogs. Among these, timolol maleate (TM), a beta-blocker, can be used to treat many types of glaucoma. Furthermore, TM does not cause blood–aqueous barrier breakdown, which induces plasma protein leakage, unlike other topical antiglaucoma drugs [7,8,9]. Therefore, TM is one of the most widely used antiglaucoma eye drops in combination with other drugs to treat both canine and human patients. TM reduces IOP by decreasing the production of the aqueous humor and blocking the beta-adrenergic receptor in ciliary body nonpigmented epithelial cells [10,11]. Particularly in dogs, TM also causes miosis [11,12,13,14,15]. As for other local effects, TM affects contralateral IOP and pupil size (PS) in dogs [11,12,13]. However, systemically absorbed TM can induce or exacerbate several adverse outcomes, such as bradycardia, hypotension, and bronchial asthma [13,14,16,17].

Nearly all water-soluble eye drops, including TM, have difficulty penetrating the hydrophobic corneal epithelium, owing to which their bioavailability is <5% [18,19,20]. The excess ophthalmic solution rapidly flows into the nasolacrimal duct and may enter the systemic circulation [18,19,20]. Several researchers in the field of ophthalmology have attempted to develop drug delivery systems (DDS) such as nanocarriers, hydrogels, and drug-absorbed contact lenses to improve the bioavailability of ophthalmic preparations [18,19,21,22,23,24,25]. While these strategies have potency in improving ocular bioavailability, they also have some drawbacks. Hydrogels prolong the drug contact time to the ocular surface, but their high viscosity may lead to ocular discomfort or irritation [26,27]. Drug-absorbed contact lenses must be retained on the corneal surface, but the shorter retention time is a major concern [28]. On the other hand, a fixed combination with a nanocarrier is a simplistic method of improving its ocular bioavailability and can be used in the same way as commercial eyedrops. Therefore, in this study, we focused on nanocarriers.

Previously, there were a few papers that focused on nanocarrier–TM fixed combinations, and one of them is our previous report applying them to rats [18]. Magnesium hydroxide, an alkaline earth metal hydroxide insoluble in water, is used for medications such as antacids and laxatives. Our previous study revealed that magnesium hydroxide nanoparticles (nMH) expand the intracellular space of the corneal epithelium, thereby increasing the corneal penetration of fixed-combined drugs such as TM, carteolol, and sericin, which improved their bioavailability in a rat model [18,19,22]. These results suggest that a fixed combination of topical nMH–TM could have greater potency and efficacy than TM alone. To our knowledge, there are no studies of the application of nanoparticle-based DDS including nMH to antiglaucoma eyedrops in dogs. This is the first investigation of the ocular hypotensive effect of the fixed combination of a nanocarrier and TM in healthy dogs.

This study aimed to investigate whether the fixed combination with nMH improves the ocular hypotensive effect of TM and affects PS, heart rate (HR), and mean arterial pressure (MAP) in clinically healthy dogs.

## 2. Materials and Methods

### 2.1. Animals

Five clinically healthy beagles with an average age and weight of 1.6 ± 0.1 years and 11.4 ± 0.7 kg, respectively, were used. Prior to experimentation, all dogs underwent complete ophthalmic examinations, which included Schirmer’s tear test 1 (Schirmer Tear Production Measuring Strips, AYUMI Pharmaceutical Corporation, Tokyo, Japan), fluorescein staining (FLURORES Ocular Examination Test Paper 0.7 mg, AYUMI Pharmaceutical Corporation, Tokyo, Japan), applanation tonometry (Tono-Pen AVIA Vet, Reichert Inc., Depew, NY, USA) after receiving the administration of topical 0.4% oxybuprocaine (Benoxil, Santen Pharmaceutical Co., Ltd., Osaka, Japan), slit-lamp biomicroscopy (Kowa SL-15, Kowa Co., Ltd., Tokyo, Japan), and indirect ophthalmoscopy (Volk 20D lens, Volk Optical Inc., Mentor, Ohio). The dogs were housed in individual cages under controlled environmental conditions (light phase from 7 a.m. to 7 p.m., and dark phase from 7 p.m. to 7 a.m.). They were cared for in accordance with the guidelines of the Animal Care and Use Committee of Kitasato University (approval no. 19-048).

### 2.2. Drugs

Regarding TM, commercially available 0.5% TM (Timoptol 0.5%, Santen Pharmaceutical Co., Ltd., Osaka, Japan) was used. Both the 0.01% and 0.1% nMH–TM fixed combination treatments, which contained 0.01% or 0.1% nMH, respectively, along with 0.5% TM, 0.5% methyl cellulose (MC), 0.5% D-mannitol, 5% hydroxypropyl-β-cyclodextrin (HPβCD), and 0.005% benzalkonium chloride (BAC), were prepared as per the instructions in our previous study [18,23]. Briefly, 0.02% or 0.2% MH was dispersed in saline containing 1% MC, 1% D-mannitol, 10% HPβCD, and 0.01% BAC, and the mixture was crushed using zirconia beads and a bead mill. After that, 0.02% or 0.2% nMH solution was diluted 1:1 with 1% TM solution. The properties of 0.02% and 0.2% nMH solutions are noted in Table S1.

### 2.3. Experimental Design

This study was performed as a single-masked, crossover study including four study periods to evaluate the following four different drugs in the same five dogs: saline, TM, 0.01% nMH–TM, and 0.1% nMH–TM. During each study period, all dogs were administered the same drug. As previously reported, an acclimation period of at least 5 days is recommended in IOP examination [29]. We also instituted IOP measurements for 5 days prior to the study to acclimate the dogs to this procedure. Each study period consisted of 12-day continuous experiments. None of the drugs were administered to establish the baseline from day 1 (D1) to D5. Furthermore, from D6 to D12 of the treatment phase, the dogs were administered 50 µL of one of the four treatments in one eye twice daily at 9 a.m. and 9 p.m., 30 min after measuring the IOP, PS, HR, and MAP. The treated eye for each dog was chosen at random. The contralateral eye was not treated. Between each study period, a withdrawal period of a minimum of 28 days was implemented to allow for the previous drug to leave the animal’s system and for the recorded parameters to return to baseline levels.

### 2.4. Measurements

The IOP, PS, HR, and MAP measurements were recorded three times daily at 9 a.m., 3 p.m., and 9 p.m. The mean daily value was defined as the mean value of measurements across these three time points. During the study period, three individual IOP measurements with a standard deviation (SD) ≤ 5% were recorded at each time point, and a single reading for each time point was calculated by averaging the three measurements. PS was measured with a digital caliper. The 1 min count of HR was measured with a stethoscope. MAP was also measured three times at each time point with an oscillometric hematomanometer (BP-608 Evolution II, Veterinary version, Fukuda Colin Co., Ltd., Tokyo, Japan), and the mean was calculated in the same way as for IOP. During the MAP measurement, the dogs were gently restrained in ventral recumbency, and a cuff with the width of 30–40% of the circumference of the cuff site was placed on the tail following the previously described guideline [30]. Examinations, including ocular irritation, Schirmer’s tear test 1, fluorescein staining, and fecal characteristics, were performed on D1 and D12 following the guidelines of a previous study [31,32].

### 2.5. Statistical Analysis

All data are presented as the mean ± SD. The obtained data from D1 to D5 of IOP, PS, HR, and MAP were statistically analyzed for each drug and day using analysis of variance (ANOVA) for repeated measures. Changes from the baseline of the overall mean values from D6 to D12 of IOP, PS, HR, and MAP were statistically analyzed for each drug using one-way ANOVA. Changes from the baseline of IOP were further analyzed for each drug at each of the three measurement time points using one-way ANOVA. The mean value from D1 to D5 was considered the baseline. When significant differences were detected in ANOVA, post hoc multiple comparisons were conducted using the Bonferroni method. All statistical analyses were performed using EZR v. 1.54 (Saitama Medical Center, Jichi Medical University, Saitama, Japan) [33]. Statistical significance was indicated by *p* < 0.05 for all values.

## 3. Results

### 3.1. Baseline Establishment

**Figure 1:**
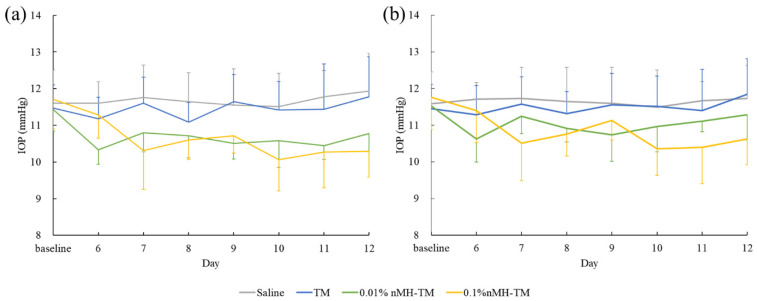
Mean daily IOP. (**a**) Treated eye; (**b**) contralateral eye. Data are presented as mean ± standard deviation. IOP; intraocular pressure, TM; 0.5% timolol maleate, 0.01% nMH–TM; 0.01% magnesium hydroxide nanoparticle–0.5% timolol maleate fixed combination, 0.1% nMH–TM; 0.1% magnesium hydroxide nanoparticle–0.5% timolol maleate fixed combination.

**Figure 2:**
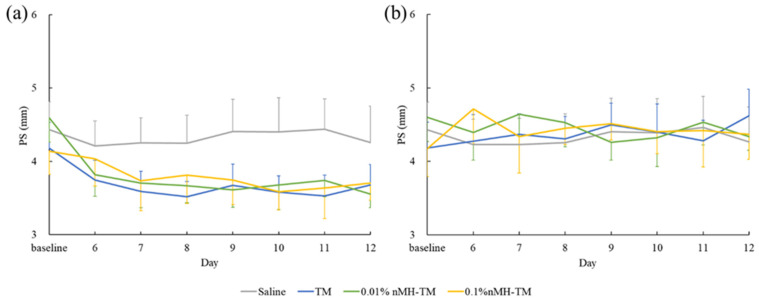
Mean daily PS. (**a**) Treated eye; (**b**) contralateral eye. Data are presented as mean ± standard deviation. PS; pupil size, TM; 0.5% timolol maleate, 0.01% nMH–TM; 0.01% magnesium hydroxide nanoparticle–0.5% timolol maleate fixed combination, 0.1% nMH–TM; 0.1% magnesium hydroxide nanoparticle–0.5% timolol maleate fixed combination.

**Figure 3:**
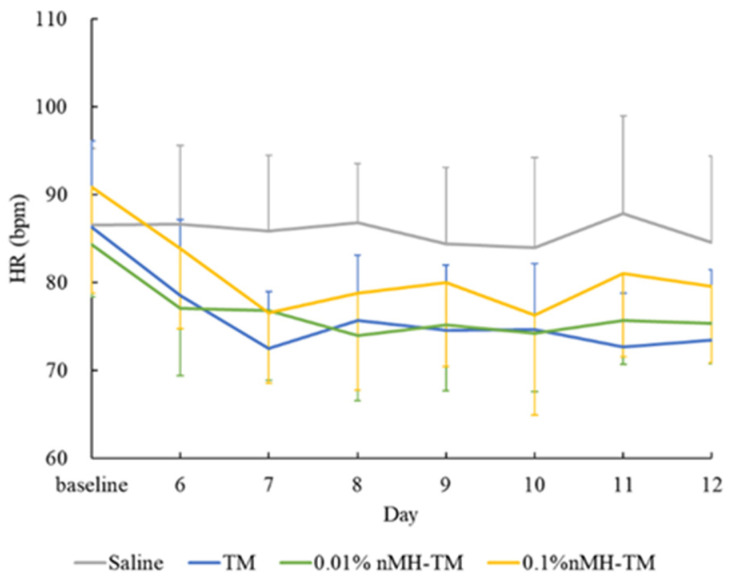
Mean daily HR. Data are presented as mean ± standard deviation. HR; heart rate, TM; 0.5% timolol maleate, 0.01% nMH–TM; 0.01% magnesium hydroxide nanoparticle–0.5% timolol maleate fixed combination, 0.1% nMH–TM; 0.1% magnesium hydroxide nanoparticle–0.5% timolol maleate fixed combination.

**Figure 4:**
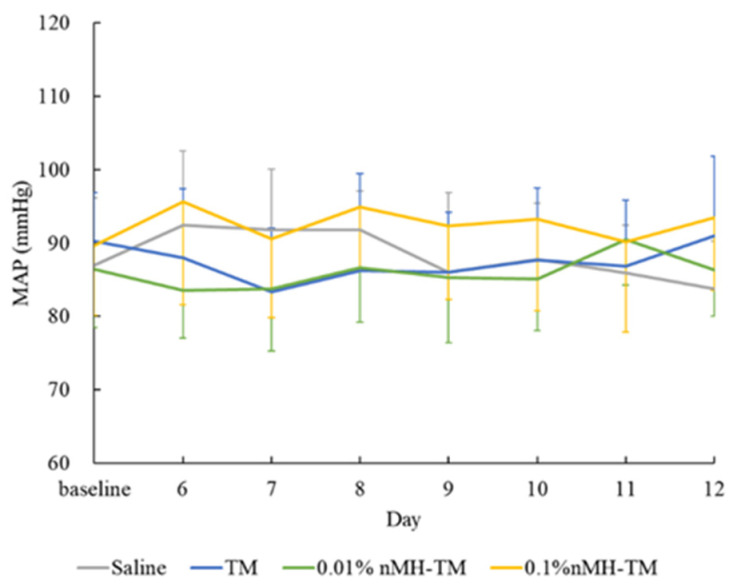
Mean daily MAP. Data are presented as mean ± standard deviation. MAP; mean arterial pressure, TM; 0.5% timolol maleate, 0.01% nMH–TM; 0.01% magnesium hydroxide nanoparticle–0.5% timolol maleate fixed combination, 0.1% nMH–TM; 0.1% magnesium hydroxide nanoparticle–0.5% timolol maleate fixed combination.

There were no significant differences in all parameters in terms of the study period (IOP of treated eye, *p* = 0.948, and IOP of contralateral eye, *p* = 0.946; PS of treated eye, *p* = 0.237, and PS of contralateral eye, *p* = 0.293; HR *p* = 0.788; and MAP *p* = 0.892) and time of day (IOP of treated eye, *p* = 0.192, and IOP of contralateral eye, *p* = 0.750; PS of treated eye, *p* = 0.189, and PS of contralateral eye, *p* = 0.247; HR, *p* = 0.721; and MAP *p* = 0.061) from D1 to D5 (Table 1, Table 2, Table 3 and Table 4 and Figure 1, Figure 2, Figure 3 and Figure 4).

### 3.2. Treatment Phase

#### 3.2.1. IOP

The mean ± SD daily IOPs for the treated eyes are summarized in Figure 1a. Changes from the baseline in the overall mean IOP and the IOP at each time point for the treated eyes are summarized in Table 5. In the treated eyes, TM did not affect the IOP until the treatment phase. The difference in the overall mean IOP from baseline in the TM-treated dogs was 0.0 ± 0.2 mmHg (ranging from −0.4 to 0.3 mmHg). This was not significantly different compared with saline-treated dogs (*p* = 1). The IOP was slightly reduced from baseline (−0.6 ± 0.4 mmHg (ranging from −1.3 to −0.2 mmHg)) at 9 p.m., but it was not significant compared with that of saline-treated dogs (*p* = 0.286). However, 0.01% nMH–TM consistently affected the IOP until the treatment phase. The change in the overall mean IOP from the baseline of the 0.01% nMH–TM-treated dogs (−0.8 ± 0.3 mmHg (from −1.2 to −0.4 mmHg)) was significantly lower than that of saline-treated dogs (*p* = 0.005) and TM-treated dogs (*p* = 0.012). Based on the diurnal variation, a significant IOP decline from the baseline of 0.01% nMH–TM-treated dogs was observed from 9 a.m. (−0.9 ± 0.4 mmHg (from −1.6 to −0.5 mmHg)) to 3 p.m. (−1.0 ± 0.1 mmHg (from −1.1 to −0.8 mmHg)) when compared to that of saline-treated dogs (both *p* = 0.013) and TM-treated dogs (*p* = 0.002, 9 a.m. and *p* < 0.001, 3 p.m.), but it was not significant at 9 p.m. (−0.7 ± 0.6 mmHg (from −1.7 to 0.3 mmHg), *p* = 0.232, saline-treated dogs, and *p* = 1, TM-treated dogs). For the 0.1% nMH–TM-treated dogs, the IOP was consistently reduced until the treatment phase. The difference in the overall mean IOP from baseline was −1.2 ± 0.4 mmHg (from −1.6 to −0.4 mmHg), which was significantly lower than that of saline-treated dogs and TM-treated dogs (both *p* < 0.001). The IOP reduction from baseline was statistically significant compared to saline-treated dogs at 9 a.m. (−1.1 ± 0.4 mmHg (from −1.6 to −0.6 mmHg); *p* = 0.030), at 3 p.m. (−1.2 ± 0.6 mmHg (from −1.8 to 0.0 mmHg); *p* = 0.002), and at 9 p.m. (−1.3 ± 0.5 mmHg (from −2.0 to −0.6 mmHg); *p* = 0.003). The IOP reduction of 0.1% nMH–TM-treated dogs was also significantly lower than that of TM-treated dogs at 9 a.m. (*p* = 0.004) and at 3 p.m. (*p* < 0.001), but it was not significant at 9 p.m. (*p* = 0.246). Significant differences between 0.01% and 0.1% nMH–TM-treated dogs were not seen at any time points (*p* = 1, 9 a.m. and 3 p.m.; *p* = 0.302, 9 p.m.; and *p* = 0.686, overall mean).

The mean ± SD daily IOPs for the contralateral eyes are summarized in Figure 1b. Changes from baseline in the overall mean IOP and at each time point for the contralateral eyes are presented in Table 6. In the contralateral eyes, each drug-treated dog showed a similar change in IOP to the treated eye. The difference in the overall mean IOP from baseline in the TM-treated dogs was 0.0 ± 0.2 mmHg (from −0.2 to 0.4 mmHg), which was not significantly different compared with saline-treated dogs (*p* = 1). The IOP was slightly reduced from baseline (−0.5 ± 0.4 mmHg (from −1.3 to −0.2 mmHg)) at 9 p.m., but it was not significant compared with that of saline-treated dogs (*p* = 0.602). Both 0.01% and 0.1% nMH–TM-treated dogs had a significantly reduced overall mean IOP from baseline (−0.5 ± 0.2 mmHg (from −0.9 to −0.2 mmHg): 0.01% nMH–TM, and −1.0 ± 0.4 mmHg (from −1.5 to −0.4 mmHg): 0.1% nMH–TM) compared with saline-treated dogs (*p* = 0.022, 0.01% nMH–TM, and *p* < 0.001, 0.1% nMH–TM) and TM-treated dogs (*p* = 0.012, 0.01% nMH–TM, and *p* < 0.001, 0.1% nMH–TM). Based on the diurnal variation, 0.01% nMH–TM-treated dogs also had a significantly reduced IOP from 9 a.m. (−0.7 ± 0.3 mmHg (from −1.2 to −0.3 mmHg)) to 3 p.m. (−0.8 ± 0.2 mmHg (from −1.2 to −0.5 mmHg)) compared with that of saline-treated dogs (*p* = 0.043, 9 a.m., and *p* = 0.012, 3 p.m.) and TM-treated dogs (*p* = 0.028, 9 a.m., and *p* < 0.001, 3 p.m.), but at 9 p.m., the IOP reduction (−0.2 ± 0.5 mmHg (from −1.0 to 0.3 mmHg)) was not significant (both *p* = 1). The IOP decline in 0.1% nMH–TM-treated dogs from baseline was statistically significant at all time points (−1.0 ± 0.3 mmHg (from −1.4 to −0.5 mmHg), 9 a.m.; −1.0 ± 0.6 mmHg (from −1.5 to 0.2 mmHg), 3 p.m.; and −1.1 ± 0.5 mmHg (from −1.7 to −0.5 mmHg), 9 p.m.) compared with that of saline-treated dogs (*p* = 0.002, 9 a.m.; *p* = 0.005, 3 p.m.; and *p* = 0.009, 9 p.m.). The IOP reduction of 0.1% nMH–TM-treated dogs was also statistically significant compared with that of TM-treated dogs at 9 a.m. (*p* = 0.001) and at 3 p.m. (*p* < 0.001), but it was not statistically significant at 9 p.m. (*p* = 0.339). A significant difference between 0.01% and 0.1% nMH–TM-treated dogs was observed at 9 p.m. (*p* = 0.028).

#### 3.2.2. PS

The mean ± SD daily PSs for the treated eyes are shown in Figure 2a. In the treated eyes, a consistent PS reduction was observed until the treatment phase in TM-treated and 0.01% and 0.1% nMH–TM-treated dogs. The differences in the overall mean PS from baseline in the TM-treated and 0.01% and 0.1% nMH–TM-treated dogs were −0.6 ± 0.2 mm (from −0.8 to −0.3 mm), −0.9 ± 0.1 mm (from −1.1 to −0.8 mm), and −0.4 ± 0.2 mm (from −0.6 to −0.2 mm), respectively. Both TM-treated and 0.01% nMH–TM-treated dogs showing a reduction in PS values were statistically significant compared with saline-treated dogs (both *p* < 0.001). In contrast, PS reduction in 0.1% nMH–TM-treated dogs was not statistically significant compared to that of saline-treated dogs (*p* = 0.109). The miotic effect of 0.01% nMH–TM was greater than that of TM (*p* = 0.022) and 0.1% nMH–TM (*p* < 0.001).

The mean ± SD daily PSs for the contralateral eyes are summarized in Figure 2b. In the contralateral eyes, none of the four drugs showed a consistent effect on PS. The differences in the overall mean PS from baseline in the TM-treated and 0.01% and 0.1% nMH–TM-treated dogs were 0.2 ± 0.2 mm (from 0.0 to 0.5 mm), −0.2 ± 0.1 mm (from −0.3 to 0.0 mm), and 0.3 ± 0.1 mm (from 0.2 to 0.4 mm), respectively. TM-treated and 0.1% nMH–TM-treated dogs significantly increased the overall mean PS from baseline compared with saline-treated dogs (*p* = 0.017, TM-treated dogs, and *p* = 0.003, 0.1% nMH–TM-treated dogs) and 0.01% nMH–TM-treated dogs (*p* = 0.004, TM-treated dogs, and *p* < 0.001, 0.1% nMH–TM-treated dogs).

#### 3.2.3. HR

The mean ± SD daily HRs are presented in Figure 3. TM-treated and 0.01% and 0.1% nMH–TM-treated dogs tended to have a consistently reduced HR until the treatment phase. The differences in the overall mean HR from baseline in the TM-treated and 0.01% and 0.1% nMH–TM-treated dogs were −11.7 ± 4.1 bpm (from −18.1 to −5.3 bpm), −8.8 ± 1.0 bpm (from −10.2 to −7.0 bpm), and −11.4 ± 8.2 bpm (from −22.6 to −4.4 bpm), respectively. The HR reductions in TM-treated and 0.1% nMH–TM-treated dogs were statistically significant compared with saline-treated dogs (*p* = 0.026, TM-treated dogs, and *p* = 0.031, 0.1% nMH–TM-treated dogs). Alternatively, the HR reduction from baseline of 0.01% nMH–TM-treated dogs was not statistically significant compared with saline-treated dogs (*p* = 0.164). There was no significant difference in the HR reduction between TM-treated and 0.01% and 0.1% nMH–TM-treated dogs (all *p* = 1).

#### 3.2.4. MAP

The mean ± SD daily MAPs are provided in Figure 4. Dogs treated with each of the four drugs did not show consistent changes in MAP until the treatment phase. There was no statistical difference between any of the four drugs (*p* = 0.105).

### 3.3. Adverse Effects

During the 5 days of pretreatment, 7 days of treatment, and at least 28 days of the interval period, there were no adverse effects for any of the four drugs based on the evaluation of ocular irritation, Schirmer’s tear test 1, fluorescein staining scores, and fecal characteristics (data not shown).

## 4. Discussion

In this study, we verified the effects of nMH–TM compared with TM in healthy dogs. Our results indicate that IOP was significantly reduced in both 0.01% and 0.1% nMH–TM-treated dogs compared with the saline- and TM-treated dogs. In contrast, the PS, HR, and BP of both 0.01% and 0.1% nMH–TM-treated dogs were almost the same as those of the TM-treated dogs.

Our data reveal that nMH–TM significantly reduced the IOP in dogs compared with TM. TM is known to have a dose-dependent ocular hypotensive effect in dogs [10]. Our previous study revealed that nMH enhanced the corneal permeability of ophthalmic solutions by expanding the corneal intracellular space, thereby increasing the ocular hypotensive effects of fixed-combined TM in a rabbit model [18,19]. Our results indicate that the canine ocular hypotensive effect of fixed-combined TM was improved by nMH, likely owing to the enhanced intraocular penetration of TM.

The ocular hypotensive effect of 0.1% nMH–TM was not significantly different from that of 0.01% nMH–TM. Our previous study demonstrated that there is no dose-dependent effect of nMH in the improvement of transcorneal penetration in rabbits [18]. Our study also suggests that 0.01% nMH may be sufficient to improve the ocular hypotensive effect of fixed-combined TM in healthy dogs.

Based on our results, TM did not have an ocular hypotensive effect in healthy dogs. At present, there have been many studies on the IOP-lowering effect of TM in healthy dogs [10,11,13,16]. This study indicates that a single eye drop of 0.5% TM reduces IOP by 16.1%, and the maximum IOP-lowering effect was observed within 2 to 4 h of topical administration [11]. A multiple daily dose study also demonstrated that 0.5% TM maximally reduced IOP by 27.1% in a 7-day treatment [13]. Another report noted that a once daily dose of 0.25% and 0.5% TM was ineffective to reduce daily IOP [10]. An additional study also described that twice daily 0.5% TM did not cause a significant decrease in IOP [16]. One of the reasons for these inconsistent results is thought to be the length of the acclimation time. A previous study indicated that at least a 5-day acclimation period is recommended to minimize the influence of patient-related factors such as stress on IOP measurements in dogs [29]. While one report did not describe the length of acclimation, and another included 12 h of acclimation [11,13], we instituted 5 days of IOP measurements to acclimate the dogs prior to the study; thereby, our results do not show any relevance of the study period and time of day during each baseline establishment phase. Another possibility of these conflicting results is differences in the measurement time points. Previous studies have shown that IOP follows a circadian rhythm in dogs and humans [6,34,35,36,37]. Therefore, a single-measurement approach might not have adequately demonstrated the effects of TM on IOP [34,35]. In consideration of this issue, 24 h monitoring of IOP has been attempted for the management of IOP [35,38,39]. In our study, multiple measurements were taken during the day, and the changes from the baseline value of the overall mean IOP and IOP at each time point were calculated and analyzed. Our results show that IOP in the TM-treated dogs did not significantly reduce compared with that of the saline-treated dogs at any time point. Based on these results, twice daily administration of 0.5% TM might be insufficient for a consistent reduction in IOP in healthy dogs.

Our results show that nMH enhanced the ocular hypotensive effect of TM in the contralateral eye, whereas nMH did not enhance the contralateral miotic effect of TM. TM also induced ocular hypotensive and miotic effects on the contralateral eye in dogs [11,12,13]. The mechanisms of these actions are unclear, but the most widely accepted mechanism is systemic absorption through the nasolacrimal duct with flow into the contralateral eye via the plasma [15]. However, the concentration of TM in the plasma when 0.5% TM is topically administered is approximately 300 times lower than that of the aqueous humor [40,41]. A low concentration of TM may be insufficient for inducing ocular hypotension and miosis in the contralateral eye. Furthermore, if contralateral ocular hypotension is caused by TM flowing into the contralateral eye via the plasma, then TM would also affect the iris sphincter muscle, meaning the contralateral eye must cause miosis in dogs. Other possible mechanisms proposed for the contralateral effects are the centrally mediated effect of a systemically absorbed drug and a consensual reaction related to changes in IOP in the treated eye [15,42]. Our results of a contralateral ocular hypotensive effect might have been caused by these mechanisms.

In our experiment, there were no ocular or systemic serious adverse effects during the 7-day treatment or during the 28-day interval periods. The systemic side effects of topically applied TM, such as bradycardia and hypotension, are related to systemic absorption from the nasolacrimal duct in both dogs and humans [14,40]. The fixed combinations of nMH did not exacerbate the bradycardia effect of TM. None of the drugs caused or exacerbated hypotension, either. Our data suggest that nMH does not increase the systemic absorption of fixed-combined TM from the nasolacrimal duct. Regarding nMH, our previous report revealed that nMH and an nMH fixed-combined drug did not affect corneal wound healing time or produce corneal toxicity in rat and rabbit models [18,19]. Additionally, 50 µL of topically administered 0.1% nMH–TM contains 50 µg of MH. This dosage is <1/1000th of that orally used as an antacid or laxative. For the above-mentioned reasons, nMH–TM is unlikely to cause serious adverse effects.

This study has some limitations. First, we used clinically healthy dogs in this study. However, ocular hypotension due to topically administered TM is more marked in glaucomatous dogs than in healthy dogs [10]. The effect of topically applied nMH–TM in glaucomatous dogs may be different from that in healthy dogs. Second, we did not measure any parameters at nighttime. It is known that the ocular hypotensive effect of TM is less effective when the IOP is lower at night than during the day in humans [6,36]. The canine IOP circadian profile is similar to that of humans [37]. Therefore, the ocular hypotensive effect of nMH–TM may show different effects at night than during the day.

## 5. Conclusions

Topical administration of 0.01% and 0.1% nMH–TM significantly reduced IOP compared with that of TM and produced minimal side effects in healthy dogs. This result suggests that nMH improved the bioavailability of fixed-combined TM. Furthermore, 0.01% and 0.1% nMH–TM may be more effective than TM alone as a canine ocular hypotensive drug. Further study to investigate these effects in glaucomatous dogs is required to verify our findings.

## Figures and Tables

**Table 1:** Mean daily IOP in the baseline phase.

Drug	Eye	D1	D2	D3	D4	D5
Saline	Treated eye	11.5 ± 1.1	11.6 ± 0.8	11.7 ± 0.9	11.3 ± 0.9	11.8 ± 1.0
	Contralateral eye	11.6 ± 1.0	11.8 ± 0.8	11.5 ± 1.0	11.3 ± 0.9	11.7 ± 1.0
TM	Treated eye	11.8 ± 0.6	11.2 ± 0.7	11.3 ± 0.9	11.5 ± 0.9	11.6 ± 0.7
	Contralateral eye	11.8 ± 0.6	11.2 ± 0.8	11.4 ± 0.8	11.3 ± 0.7	11.4 ± 0.9
0.01% nMH–TM	Treated eye	11.3 ± 0.8	11.5 ± 0.6	11.7 ± 0.7	11.4 ± 0.6	11.2 ± 0.7
	Contralateral eye	11.5 ± 0.8	11.5 ± 0.5	11.7 ± 0.6	11.6 ± 0.6	11.4 ± 0.7
0.1% nMH–TM	Treated eye	11.7 ± 0.8	11.7 ± 1.1	12.0 ± 1.0	11.3 ± 0.6	11.8 ± 0.8
	Contralateral eye	11.7 ± 0.9	11.7 ± 1.0	12.3 ± 1.1	11.2 ± 0.7	11.9 ± 0.9

Data are presented as mean ± standard deviation (mmHg). IOP, intraocular pressure; TM, 0.5% timolol maleate; 0.01% nMH–TM, 0.01% magnesium hydroxide nanoparticle–0.5% timolol maleate fixed combination; 0.1% nMH–TM, 0.1% magnesium hydroxide nanoparticle–0.5% timolol maleate fixed combination.

**Table 2:** Mean daily PS in the baseline phase.

Drug	Eye	D1	D2	D3	D4	D5
Saline	Treated eye	4.4 ± 0.4	4.4 ± 0.4	4.5 ± 0.4	4.5 ± 0.3	4.4 ± 0.5
	Contralateral eye	4.4 ± 0.4	4.4 ± 0.4	4.5 ± 0.3	4.5 ± 0.3	4.4 ± 0.5
TM	Treated eye	4.0 ± 0.4	4.2 ± 0.3	4.1 ± 0.3	4.2 ± 0.3	4.4 ± 0.4
	Contralateral eye	4.0 ± 0.4	4.2 ± 0.3	4.1 ± 0.3	4.2 ± 0.3	4.4 ± 0.4
0.01% nMH–TM	Treated eye	4.7 ± 0.5	4.3 ± 0.3	4.5 ± 0.4	4.6 ± 0.3	4.8 ± 0.3
	Contralateral eye	4.7 ± 0.4	4.3 ± 0.3	4.5 ± 0.4	4.6 ± 0.3	4.8 ± 0.2
0.1% nMH–TM	Treated eye	4.2 ± 0.3	4.4 ± 0.6	4.0 ± 0.3	4.0 ± 0.3	4.1 ± 0.4
	Contralateral eye	4.3 ± 0.3	4.4 ± 0.6	4.1 ± 0.3	4.1 ± 0.4	4.1 ± 0.4

Data are presented as mean ± standard deviation (mm). PS, pupil size; TM, 0.5% timolol maleate; 0.01% nMH–TM, 0.01% magnesium hydroxide nanoparticle–0.5% timolol maleate fixed combination; 0.1% nMH–TM, 0.1% magnesium hydroxide nanoparticle–0.5% timolol maleate fixed combination.

**Table 3:** Mean daily HR in the baseline phase.

Drug	D1	D2	D3	D4	D5
Saline	87.1 ± 7.7	85.7 ± 9.3	88.1 ± 10.2	87.3 ± 8.6	84.4 ± 8.6
TM	85.9 ± 10.1	87.5 ± 12.5	83.3 ± 8.7	84.8 ± 11.2	90.0 ± 7.5
0.01% nMH–TM	86.8 ± 4.5	83.5 ± 8.3	84.3 ± 7.9	82.7 ± 5.4	84.1 ± 5.4
0.1% nMH–TM	91.7 ± 9.9	92.5 ± 14.9	90.5 ± 15.6	90.1 ± 10.3	89.5 ± 12.9

Data are presented as mean ± standard deviation (bpm). HR, heart rate; TM, 0.5% timolol maleate; 0.01% nMH–TM, 0.01% magnesium hydroxide nanoparticle–0.5% timolol maleate fixed combination; 0.1% nMH–TM, 0.1% magnesium hydroxide nanoparticle–0.5% timolol maleate fixed combination.

**Table 4:** Mean daily MAP in the baseline phase.

Drug	D1	D2	D3	D4	D5
Saline	90.8 ± 10.4	86.3 ± 9.0	88.3 ± 12.0	85.6 ± 9.3	84.0 ± 7.2
TM	93.0 ± 6.6	89.2 ± 4.1	89.3 ± 10.5	87.0 ± 6.8	92.8 ± 7.8
0.01% nMH–TM	86.7 ± 8.7	84.4 ± 9.8	86.0 ± 5.6	90.0 ± 9.6	85.4 ± 8.1
0.1% nMH–TM	90.4 ± 10.3	86.6 ± 9.6	90.7 ± 8.3	87.8 ±10.6	92.8 ± 10.8

Data are presented as mean ± standard deviation (mmHg). MAP, mean arterial pressure; TM, 0.5% timolol maleate; 0.01% nMH–TM, 0.01% magnesium hydroxide nanoparticle–0.5% timolol maleate fixed combination; 0.1% nMH–TM, 0.1% magnesium hydroxide nanoparticle–0.5% timolol maleate fixed combination.

**Table 5:** Measured IOP of baseline and treatment phase in the treated eye.

Drug	Parameter	Baseline				Treatment Phase			
		9 A.M.	3 P.M.	9 P.M.	Overall Mean	9 A.M.	3 P.M.	9 P.M.	Overall Mean
Saline	ΔIOP (mmHg)	11.4 ± 1.1	11.7 ± 0.9	11.7 ± 0.8	11.6 ± 0.9	0.0 ± 0.4	0.2 ± 0.4	0.1 ± 0.2	0.1 ± 0.2
	IOP (mmHg)					11.4 ± 0.9	11.9 ± 1.0	11.7 ± 0.7	11.7 ± 0.8
TM	ΔIOP (mmHg)	11.6 ± 0.7	10.9 ± 0.8	11.9 ± 0.5	11.5 ± 0.6	0.0 ± 0.2	0.6 ± 0.4	−0.6 ± 0.4	0.0 ± 0.2
	IOP (mmHg)					11.5 ± 0.8	11.6 ± 0.8	11.3 ± 0.8	11.5 ± 0.8
0.01% nMH–TM	ΔIOP (mmHg)	11.6 ± 0.9	11.5 ± 0.5	11.2 ± 0.7	11.4 ± 0.6	−0.9 ± 0.4 ^a,b^	−1.0 ± 0.1 ^a,b^	−0.7 ± 0.6	−0.8 ± 0.3 ^a,b^
	IOP (mmHg)					10.7 ± 0.6	10.5 ± 0.5	10.5 ± 0.4	10.6 ± 0.4
0.1% nMH–TM	ΔIOP (mmHg)	11.4 ± 0.9	11.7 ± 0.8	12.0 ± 0.8	11.7 ± 0.8	−1.1 ± 0.4 ^a,b^	−1.2 ± 0.6 ^a,b^	−1.3 ± 0.5 ^a^	−1.2 ± 0.4 ^a,b^
	IOP (mmHg)					10.3 ± 0.6	10.5 ± 0.6	10.7 ± 0.8	10.5 ± 0.6

Data are presented as mean ± standard deviation. ^a^ *p* < 0.05 vs. saline-treated dog; ^b^ *p* < 0.05 vs. TM-treated dog. IOP, intraocular pressure; ΔIOP, changes from baseline value of IOP; TM, 0.5% timolol maleate; 0.01% nMH–TM, 0.01% magnesium hydroxide nanoparticle–0.5% timolol maleate fixed combination; 0.1% nMH–TM, 0.1% magnesium hydroxide nanoparticle–0.5% timolol maleate fixed combination.

**Table 6:** Measured IOP of baseline and treatment phase in the contralateral eye.

Drug	Parameter	Baseline				Treatment Phase			
		9 A.M.	3 P.M.	9 P.M.	Overall Mean	9 A.M.	3 P.M.	9 P.M.	Overall Mean
Saline	ΔIOP (mmHg)	11.5 ± 1.0	11.7 ± 0.9	11.6 ± 0.8	11.6 ± 0.9	0.0 ± 0.3	0.2 ± 0.4	0.0 ± 0.2	0.1 ± 0.2
	IOP (mmHg)					11.5 ± 0.8	11.9 ± 0.9	11.6 ± 0.7	11.7 ± 0.8
TM	ΔIOP (mmHg)	11.6 ± 0.7	10.9 ± 0.8	11.8 ± 0.6	11.4 ± 0.7	0.0 ± 0.2	0.6 ± 0.4	−0.5 ± 0.4	0.0 ± 0.2
	IOP (mmHg)					11.6 ± 0.7	11.6 ± 0.9	11.3 ± 0.9	11.5 ± 0.8
0.01% nMH–TM	ΔIOP (mmHg)	11.8 ± 0.8	11.6 ± 0.5	11.2 ± 0.7	11.5 ± 0.5	−0.7 ± 0.3 ^a,b^	−0.8 ± 0.2 ^a,b^	−0.2 ± 0.5	−0.5 ± 0.2 ^a,b^
	IOP (mmHg)					11.1 ± 0.6	10.8 ± 0.6	11.0 ± 0.4	11.0 ± 0.5
0.1% nMH–TM	ΔIOP (mmHg)	11.6 ± 0.9	11.7 ± 0.9	11.9 ± 1.0	11.8 ± 0.9	−1.0 ± 0.3 ^a,b^	−1.0 ± 0.6 ^a,b^	−1.1 ± 0.5 ^a,c^	−1.0 ± 0.4 ^a,b^
	IOP (mmHg)					10.6 ± 0.7	10.8 ± 0.7	10.8 ± 0.8	10.7 ± 0.6

Data are presented as mean ± standard deviation. ^a^ *p* < 0.05 vs. saline-treated dog; ^b^ *p* < 0.05 vs. TM-treated dog; ^c^ *p* < 0.05 vs. 0.01% nMH–TM-treated dog. IOP, intraocular pressure; ΔIOP, changes from baseline value of IOP; TM, 0.5% timolol maleate; 0.01% nMH–TM, 0.01% magnesium hydroxide nanoparticle–0.5% timolol maleate fixed combination; 0.1% nMH–TM, 0.1% magnesium hydroxide nanoparticle–0.5% timolol maleate fixed combination.

## Data Availability

The data presented in this study are available on request from the corresponding author.

## Supplementary Materials

The following are available online at https://www.mdpi.com/article/10.3390/vetsci8080168/s1, Table S1: Particle size distribution of 0.02% and 0.2% nMH.
