# Supplementary material for: Magnesium Hydroxide Nanoparticles Improve the Ocular Hypotensive Effect of Twice Daily Topical Timolol Maleate in Healthy Dogs
Source: Vet Sci. 2021 Aug 23;8(8):168. doi: 10.3390/vetsci8080168 (PMC8402916; doi:10.3390/vetsci8080168)
Supplement: Supplementary file 1 [file vetsci-08-00168-s001.zip › vetsci-1313503-supplementary.pdf]

Table S1. Particle size distribution of 0.02% and 0.2% nMH.

	D10 (nm)	D50 (nm)	D90 (nm)
0.02% nMH	66	98	178
0.2% nMH	61	101	172

D10, 50, and 90; The portion of particles with diameters smaller than this value is 10% , 50%, and 90%, respectively.
